# A nanoluciferase complementation-based assay for monitoring β-arrestin2 recruitment to the dopamine D_3_ receptor

**DOI:** 10.1016/j.bbrep.2025.102019

**Published:** 2025-04-18

**Authors:** Viktor Burström, Kuiying Xu, Emilio Garro-Martínez, Robert H. Mach, Kristoffer Sahlholm, Nibal Betari

**Affiliations:** a Department of Medical and Translational Biology, Wallenberg Centre for Molecular Medicine, Umeå University, 901 87, Umeå, Sweden; b Department of Radiology, Perelman School of Medicine, University of Pennsylvania, Philadelphia, PA, 19104-6323, United States; c Department of Physiology and Pharmacology, Karolinska Institutet, 171 65, Solna, Sweden

**Keywords:** Luminescence measurements, Luciferase, HEK 293 cells, G protein-coupled receptor kinase

## Abstract

Luciferase complementation assays have emerged as a simple means of monitoring receptor-effector interactions in living cells in a time-resolved manner. Here, we describe a nanoluciferase complementation assay capable of reporting on β-arrestin2 recruitment to the human dopamine D_3_ receptor (D_3_R) upon its activation in intact HEK293T cells. Using this assay in time-resolved experiments, we detect differences in arrestin response termination rates between the endogenous agonist dopamine and the synthetic D_3_R agonist FAUC-73. We also investigate the influence of exogenous GRK2 on β-arrestin2 recruitment to the D_3_R. We find that, in contrast to the D_2_R and D_4_R, the potency of dopamine to induce arrestin recruitment to D_3_R is not significantly influenced by GRK2 overexpression. In further agreement with a lack of GRK2 regulation of D_3_R signalling and again contrary to the D_2_R and D_4_R, we do not observe dopamine-induced recruitment of GRK2 to D_3_R. Conversely, dopamine concentration-dependently decreases the interaction between GRK2 and D_3_R. Additionally, we examine both the Ser-9 and Gly-9 variants of the human D_3_R, which, according to some earlier reports, differ in terms of dopamine affinity and functional potency. However, we find no difference in the concentration-response relationships between these two variants, neither when arrestin recruitment nor GRK2 interactions are studied. In summary, the present report demonstrates the utility of nanoluciferase complementation for studying D_3_R pharmacology in living cells.

## Highlights

• A luciferase assay for arrestin recruitment to the dopamine D3 receptor is described.
• Ser9Gly polymorphism in D3 does not affect dopamine potency in arrestin recruitment.
• GRK2 is not recruited to activated D3, nor does GRK2 enhance arrestin recruitment.
• Receptor-agonist-arrestin complex is less stable with FAUC-73 than with dopamine.
• Nanoluciferase complementation allows for time-resolved arrestin assays in live cells.

## 1 Introduction

Dopamine D_2_-like receptors, which comprise the dopamine D_2_ receptor (D_2_R), D_3_R, and D_4_R, are G protein-coupled receptors (GPCRs) that mainly signal via inhibitory G_i/o_ proteins, inhibiting adenylate cyclase, closing calcium channels, and opening G protein-coupled inward rectifier potassium (GIRK, also known as Kir3) channels [1]. In addition to G protein signalling, D_2_-like receptors also signal via arrestin-dependent pathways. While arrestins were originally thought of only as terminators of G protein-dependent signalling, later investigations have shown them to initiate their own signalling cascades [2,3]. In the case of the D_3_R, arrestin-dependent signalling has been found to regulate neuronal firing patterns in brain tissue [4], and to impact antipsychotic-like efficacy in rodent models [5]. G protein-coupled receptor kinases (GRKs) constitute a third class of GPCR-interacting proteins. Their best described function is to promote arrestin recruitment to activated GPCRs, frequently (but not exclusively) by phosphorylating serine and threonine residues in the intracellular regions of the receptors, thereby increasing arrestin binding affinity. Whereas arrestin recruitment to both the D_2_R and D_4_R has been shown to be enhanced by GRK2 co-expression [6], there have been conflicting reports on the role of GRKs in modulating D_3_R-arrestin interactions. Thus, some reports have suggested GRK2 coexpression to decrease basal and enhance agonist-induced D_3_R-arrestin interactions, whereas others have described no effect [[7], [8], [9]].

The D_3_R has been implicated in several psychiatric and neurological disorders including schizophrenia, substance abuse, restless legs, and Parkinson's disease. Specifically, D_3_R-selective or preferring antagonists and partial agonists have generated interest as antipsychotic, anti-abuse, and anti-dyskinetic drugs for treatment of l-DOPA-induced dyskinesia in Parkinson's disease [10,11]. Cariprazine, a partial D_2/3_R agonist with ∼10-fold higher affinity for D_3_R, has proven to be more efficacious in treating negative symptoms in schizophrenia as compared to the second-generation antipsychotic risperidone [12]. On the other hand, more efficacious D_3_R-preferring D_2/3_R agonists such as pramipexol and ropinirole are being used to treat motor symptoms of Parkinson's disease, as well as restless legs disorder. However, these agonists have been linked to impulse control disorders, a link which has been proposed to reflect the role of the D_3_R in reward-seeking behaviour [13].

Interestingly, the human D_3_R exists as two common alleles with translational consequences, thus differing by the amino acid residue at position 9 in the extracellular N-terminus of the D_3_R (Ser9Gly; rs6280). Ser-9 has an allele frequency of 0.62 in the Genome Aggregation Database (gnomAD) v.2.1.1 [14]. The less frequent allele, Gly-9, has been tied to a higher risk of impulse control disorders in Parkinson's patients [15], essential tremor [16], and tardive dyskinesia in patients treated with antipsychotics [17], among other conditions. However, later meta-analyses have cast doubt on the robustness of several of these associations [18,19], or suggested that the impact of the polymorphism may differ between ethnic populations [20]. A few functional studies have suggested differences in dopamine binding affinity and efficacy between the two variants [16,21], whereas others failed to replicate these findings [22]. While position 9 is not located within the orthosteric binding pocket, computational studies have suggested that the N-terminus folds down to form a “lid” on the extracellular surface of the receptor, potentially affecting ligand access and/or egress [23], thus providing a rationale for how this polymorphism could affect D_3_R binding characteristics. Furthermore, a nearby N-linked glycosylation site at position 12, conserved among D_2_-like receptors, suggests the possibility for an impact of Ser9Gly on maturation and trafficking of the receptor, although no such differences were reported from transfected cells [16]. While previous studies examined G protein-dependent signalling and radioligand binding affinity, arrestin recruitment by the Ser9Gly variants have yet to be studied. Here, we describe a nanoluciferase complementation assay capable of reporting on arrestin recruitment to the D_3_R upon its activation by agonists. Moreover, we use this assay to compare the potencies of dopamine at the Ser9Gly variants, as well as the relative stabilities of agonist-receptor-arrestin complexes induced by the native agonist or the synthetic ligand FAUC-73 [24].

## 2 Materials and methods

### 2.1 Molecular biology

The D_3_R–NP Ser-9 and Gly-9 constructs (NP: 'native peptide') were synthesized by Genscript (Piscataway, NJ) on a pcDNA3.1+ backbone (Thermo Fisher Scientific, Waltham, MA). These receptor constructs contained an N-terminal cleavable influenza hemagglutinin signal peptide (KTIIALSYIFCLVFA), which promotes GPCR surface expression, followed by a FLAG tag (DYKDDDDK), as well as a C-terminal linker (GNSGSSGGGGSGGGGSSG) and NP tag (GVTGWRLCERILA) [25].

Rat βarr2 (in pNBe3; Promega, Madison, WI) was N-terminally tagged with LgBiT;

VFTLEDFVGDWEQTAAYNLDQVLEQGGVSSLLQNLAVSVTPIQRIVRSGENALKIDIHVIIPYEGLSADQMAQIEEVFKVVYPVDDHHFKVILPYGTLVIDGVTPNMLNYFGRPYEGIAVFDGKKITVTGTLWNGNKIIDERLITPDGSMLFRVTINS, and was obtained from Dr. Julien Hanson (University of Liège, Belgium). Untagged human GRK2 (in pXOOM; from prof. Søren-Peter Olesen, University of Copenhagen, Denmark) was synthesized by Genscript. LgBiT was fused directly to the C-terminus of human GRK2 to create GRK2-LgBiT.

### 2.2 Luciferase complementation assay

HEK293T cells (a gift from Dr. Jonathan Gilthorpe, Umeå University, Sweden) were cultured at 37 °C with 5 % CO_2_ in 10 cm culture plates (VWR part of Avantor, Radnor, PA). The culture medium was Dulbecco's modified eagle medium (DMEM; Thermo Fisher) containing 0.01 % penicillin/streptomycin (Thermo Fisher) and 10 % FBS (Thermo Fisher). Cells were transfected at about 80 % confluency with 1 μg/dish D_3_R–NP and 1 μg/dish of either LgBiT-βarr2 or GRK2-LgBiT and, when indicated, 10 μg untagged GRK2, using linear polyethylenimine (PEI; Polysciences Inc., Valley Road Warrington, PA) [26]. The plasmid backbone (pcDNA3.1+) was added as necessary to bring the total amount of cotransfected plasmid to 20 μg/dish. 24 h after transfection, cells were resuspended in Hank's balanced salt solution (HBSS; Corning, Tewksbury, MA), counted using a TC20 cell counter (Bio-Rad, Hercules, CA), and diluted in HBSS. Each well of a flat-bottom white 96-well plate (Thermo Fisher) received 50 000 cells suspended in 100 μl HBSS. The nanoluciferase substrate furimazine (Nano-Glo live cell reagent, Promega) was diluted 1/19 in the provided reagent buffer and 10 μl of this dilution was added to each well. For concentration-response experiments, serial dilutions of dopamine (Sigma-Aldrich, St. Louis, MO) or FAUC-73 (synthesized according to published methods [24] at the Department of Radiology, University of Pennsylvania, PA) were added to the 96-well plate in octuplicates, with the control column receiving only HBSS. After agonist addition, the plate was incubated at room temperature for 15 min before reading the luminescence in a TriStar^2^ LB 942 plate reader (Berthold Technologies, Bad Wildbad, Germany), using a counting time of 10 ms/well.

For time-resolved experiments, cells were lifted off after transfection and seeded into flat-bottom white 96-well plates coated with poly-d-lysine (Sigma-Aldrich). Experiments were performed 24 h later and luminescence was repeatedly recorded from each well every 14 s with an integration time of 5 ms. Following a baseline read of 81 s, 10 μl of dopamine or FAUC-73 dissolved in HBSS were injected into each well to yield a final concentration of 1 or 100 nM, respectively. After another 147 s, 10 μl of the D_3_R antagonist SB277011A [27] (Tocris Bioscience, Bristol, UK) dissolved in DMSO and diluted in HBSS to yield a final concentration of 10 μM were injected.

### 2.3 Whole-cell enzyme-linked immunosorbent assay (ELISA)

Surface expression of D_3_R constructs on intact cells was measuring using a previously described protocol [28]. Briefly, 24 h after transfection (performed as described above), cells were resuspended in DMEM (supplemented as above) and plated at 50 000 cells/well in poly-d-lysine-coated transparent 96-well plates (Thermo Fischer). After another 24 h of culture, DMEM was aspirated, and wells were washed twice with 100 μl/well of chilled (4 °C) phosphate-buffered saline (PBS; VWR part of Avantor) containing 0.5 % (w/v) bovine serum albumin (BSA; Sigma-Aldrich). Subsequently, 50 μl of horseradish peroxidase-linked mouse anti-FLAG M2 antibody (A8592; Sigma-Aldrich) diluted 1/20000 in chilled PBS containing 1 % BSA were added to each well. After 1 h incubation at 4 °C, the antibody dilution was aspirated and wells were washed four times with chilled PBS containing 0.5 % BSA. Thereafter, 50 μl/well of the horseradish peroxidase substrate 3,3′,5,5′-Tetramethylbenzidine (T0440; Sigma-Aldrich) were added, followed by a final 20-min incubation at 37 °C. Lastly, each well received 50 μl of 2 M HCl, after which the absorbance at 450 nm was measured in the Berthold TriStar^2^ LB 942 plate reader.

### 2.4 Data analysis

Concentration–response curves were generated using GraphPad Prism 8 (GraphPad Software, San Diego, CA), fitting the following equation to data:

(1) $$Y=Bottom+\left(Top-Bottom\right)/\left(1+10^{\left(\log\;{\text{EC}}_{50}-X\right)}\right)$$

where *Y* is the response, *Bottom* is the baseline response, *Top* is the maximal response as a fraction of the mean control luminescence and *X* is the logarithm of agonist concentration. No parameters were constrained during curve fitting. The distributions of data sets were tested using the Shapiro-Wilk test. Conditions were compared using Student's paired *t*-test or two-way repeated measures ANOVA with Sidak's test for multiple comparisons in GraphPad Prism 8, testing differences in pEC_50_s or efficacy (Top – Bottom) parameters between Ser-9 and Gly-9 and in the presence or absence of exogenous GRK2, or differences in normalized response amplitude between dopamine and FAUC-73 following antagonist addition. Paired experiments were carried out on the same day with cells that had undergone the same number of passages.

## 3 Results/discussion

First, we studied dopamine-induced arrestin recruitment to the human D_3_R by coexpressing D_3_R–NP with LgBiT-β-arrestin2 in HEK293T cells. Rather than using the standard “SmBiT” as the smaller fragment to reconstitute the full enzymatic activity of the larger “LgBiT” fragment, we employed a “native peptide” (NP) fragment. Compared to SmBiT, NP has somewhat higher affinity for LgBiT and has been shown to result in higher signal-to-noise in assays monitoring G protein recruitment to the D_2_R [25]. Importantly, complementation between NP and LgBiT has been shown to be readily reversible [6,25]. The non-visual arrestins, β-arrestin1 and β-arrestin2 (also known as arrestin2 and arrestin3, respectively), are widely expressed in a multitude of cell types. Here, we chose to focus on β-arrestin2 since the closely related D_2_R has been reported to preferentially interact with this subtype in native tissue [29]. Additionally, β-arrestin2 is known to be involved in behavioural responses to dopaminergic signalling [2,30,31].

**Fig. 1:**
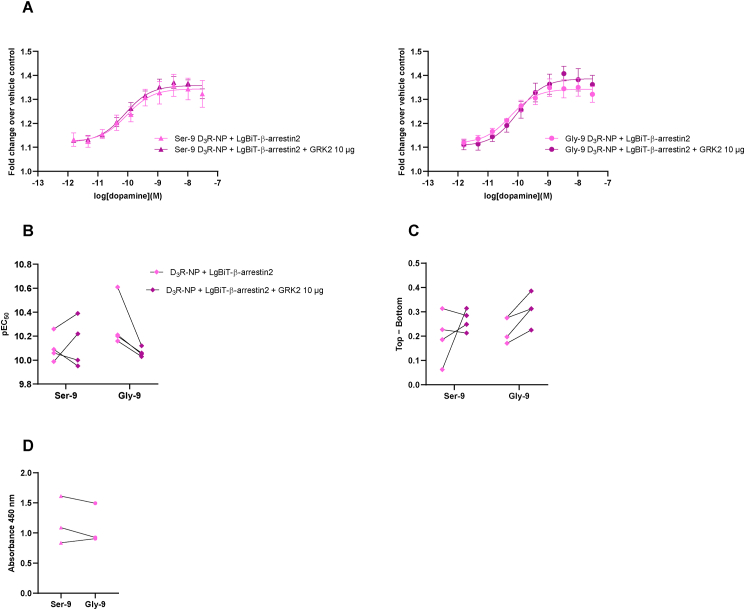
A nanoluciferase complementation assay to monitor arrestin recruitment to the D_3_R in live cells. A) Concentration-response curves for dopamine-induced luminescence increase obtained in cells transfected with Ser-9 (left) or Gly-9 (right) D_3_R–NP constructs and LgBiT-βarrestin2, with or without GRK2, as indicated. Data points represent means ± s.e.m. from four individual experiments performed in octuplicate wells. Fits of concentration-response curves to the means are shown to guide the eye. B) pEC_50_ and C) efficacy expressed as Top − Bottom parameters fitted to data from each of the four individual experiments, the means of which are shown in A. No significant differences are observed, neither between genotype (Gly-9 vs. Ser-9) nor with or without GRK2. (repeated measures 2-way ANOVA with Sidak's test for multiple comparisons). Data from four experiments for each condition (Ser-9 and Gly-9, with and without GRK2), are shown, but symbols sometimes overlap. See also Supplementary Table S1 for a tabular representation of the data, including s.e.m. D) Surface FLAG immunoreactivity on HEK293T cells expressing the FLAG-tagged Gly-9 or Ser-9 D_3_R–NP constructs. Three independent experiments were performed in octuplicate wells. No statistical differences between variants are observed (Student's paired *t*-test). See also Supplementary Table S2 for a tabular representation of the data, including s.e.m.

**Fig. 2:**
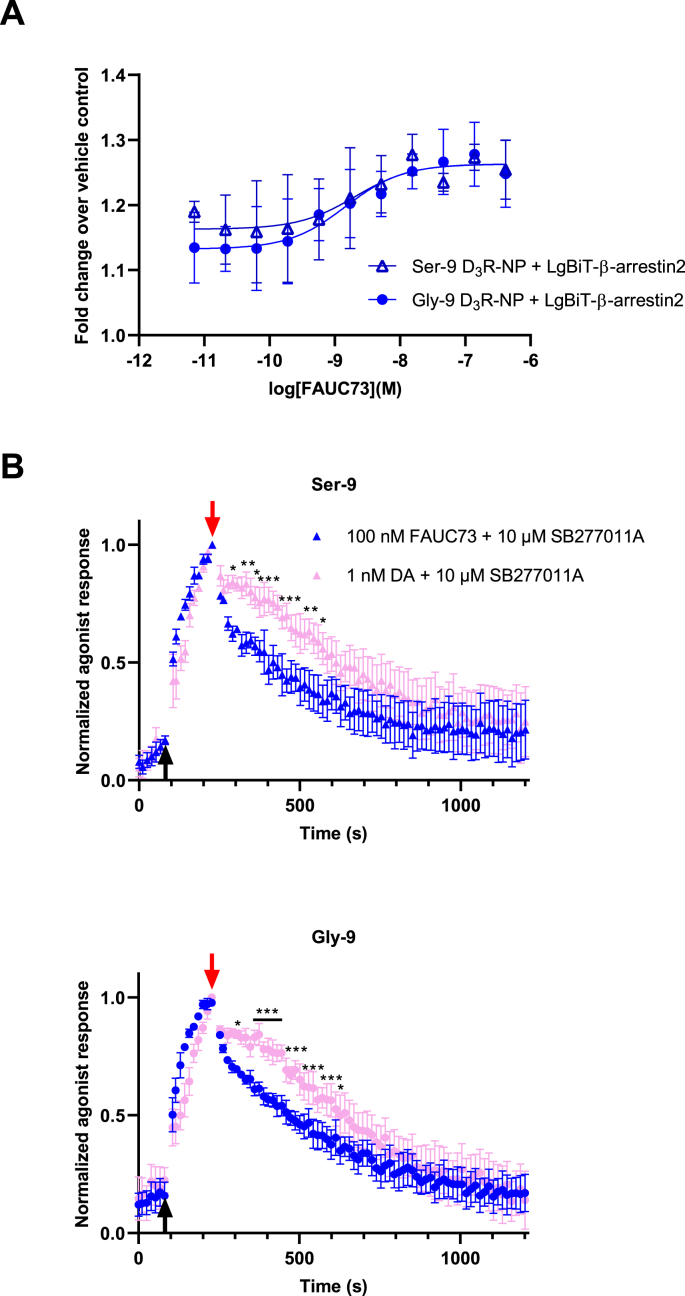
Kinetic differences between FAUC-73 and dopamine in the nanoluciferase complementation-based arrestin recruitment assay. A) Concentration-response curves for FAUC-73-induced luminescence increase obtained in cells transfected with Gly-9 or Ser-9 D_3_R–NP constructs, as indicated, and LgBiT-βarrestin2. Data points represent means ± s.e.m. from three individual experiments performed in quadruplicate wells. B) Time-resolved responses to 100 nM FAUC-73 or 1 nM dopamine (black arrow), followed by the application of 10 μM SB277011A (red arrow), in cells coexpressing Ser-9 (above) or Gly-9 (below) D_3_R-NP with LgBiT-βarrestin2. Data represent means ± s.e.m. from four individual experiments performed in octuplicate wells, normalized first to baseline and then individually to the response amplitude at the moment of antagonist application. Asterisks indicate statistically significant differences between agonist treatment. ∗, p < 0.05; ∗∗, p < 0.01; ∗∗∗, p < 0.001; repeated measures ANOVA with Time and Agonist as factors and Sidak's correction for multiple comparisons. Asterisks refer to single pairs of data points, although not all significant pairs can be labelled for clarity. Horizontal bar with asterisks in the lower (Gly-9) panel indicates a consecutive series of data point pairs where p < 0.001. At a main effects level, significances were noted for Time and for the interaction between Time and type of Agonist (i.e., FAUC-73 or dopamine). For Ser-9, F (69, 207) = 44.39, p < 0.001 for Time and F (69, 207) = 1.605, p = 0.0058 for the interaction between Time and Agonist. For Gly-9, F (69, 207) = 73.44, p < 0.001 for Time and F (69, 207) = 2.877, p < 0.001 for the interaction between Time and Agonist.

24 h after transfection, cells were lifted off and re-seeded in HBSS into 96-well plates at 50 000 cells/well. Following addition of the nanoluciferase substrate furimazine, dopamine application increased luminescence output in a concentration-dependent manner, consistent with recruitment of arrestin to the activated receptor (Fig. 1A). Importantly, we have previously demonstrated that agonist application to cells expressing LgBiT-β-arrestin2 alone does not cause changes in luminescence output [6] (see also Supplementary Fig. S1). Likewise, in cells expressing D_3_R–NP alone, luminescence (which was at background levels) was not altered by D_3_R agonist application (Supplementary Fig. S1). Experiments with Ser-9 and Gly-9, with or without exogenous GRK2, were performed in parallel and paired analyses were used to assess effects of D_3_R variant and of GRK2 overexpression. Two-way repeated measures ANOVAs were performed, testing the effects of genotype (Ser-9 vs. Gly-9) and GRK2 coexpression on pEC_50_s and on dopamine efficacy (Top − Bottom), respectively. No significant differences in pEC_50_s were observed between the Ser-9 (pEC_50_ 10.03 ± 0.26) and Gly-9 (pEC_50_ 10.25 ± 0.19) variants, nor were there any significant effects of GRK2 cotransfection on agonist potency (pEC_50_ 10.09 ± 0.14 and 10.00 ± 0.18 for Ser-9 and Gly-9 cotransfected with GRK2, respectively; Fig. 1A and B and Supplementary Table S1) at amounts of GRK2 plasmid equal to those that significantly increased dopamine potency in corresponding assays of arrestin recruitment to the D_2_R and D_4_R [6]. Neither were there any significant differences between the two variants in terms of the maximal dopamine-induced luminescence increase (Top – Bottom 0.22 ± 0.03 and 0.23 ± 0.02 for Ser-9 and Gly-9, respectively), nor did cotransfection of GRK2 have any significant effect on arrestin recruitment at the level of efficacy (Top – Bottom 0.238 ± 0.016 and 0.281 ± 0.025 for Ser-9 and Gly-9 cotransfected with GRK2, respectively; Fig. 1A, and C and Supplementary Table S1). In agreement with earlier reports [16], surface expression of the two variants was similar (Fig. 1D and Supplementary Table S2). In separate experiments, similar concentration-response relationships (pEC_50_ 8.69 ± 0.75 and 8.84 ± 0.51 for Ser-9 and Gly-9, respectively) were found for arrestin recruitment by the synthetic D_3_R agonist FAUC-73 ([24], Fig. 2A). Compared to dopamine, the responses elicited by FAUC-73 tended to be of lower amplitude (Top − Bottom 0.10 ± 0.04 and 0.13 ± 0.03 for Ser-9 and Gly-9, respectively), in agreement with reports of FAUC-73 having a lower efficacy (about 70 %) compared to the full D_3_R agonist quinpirole [32].

In GIRK activation assays, the D_3_R displays much slower response termination kinetics upon dopamine washout compared to the D_2_R and D_4_R [[33], [34], [35]]. However, the GIRK response to the synthetic D_3_R agonist FAUC-73 was reported to decay significantly faster than that of dopamine [35]. Slow GIRK response termination upon agonist washout has been suggested to correspond to slow breakdown of the receptor-G-protein-agonist complex, thus reflecting slow agonist dissociation from the receptor [[36], [37], [38], [39]]. In contrast to G protein-dependent GIRK activation, the decay kinetics of dopamine or FAUC-73-evoked arrestin responses have not been reported previously. Using 10 μM of the D_3_R antagonist SB277011A (K_i_ 11 nM) [27] to terminate agonist access to the D_3_R, the time courses of response decay, presumably reflecting breakdown of the ternary complex of receptor, agonist, and arrestin upon agonist dissociation, were compared between dopamine and FAUC-73 at concentrations 10- to 20-fold (dopamine) or 50- to 70-fold (FAUC-73) higher than the EC_50_s of the two agonists (1 nM and 100 nM, respectively; Fig. 2B). Significant differences in the deactivation time courses were observed between the two agonists, with the FAUC-73-induced response decaying faster than that evoked by dopamine. Based on the present observations, we suggest that dopamine dissociates slower than FAUC-73 from the D_3_R also when the receptor is complexed with arrestin instead of G protein. To enable comparison of the decay time courses, data sets were normalized individually both to baseline and to the response amplitude at the moment of antagonist application (Fig. 2B). For the corresponding data normalized only to baseline, please see Supplementary Fig. S2. Notably, the agonist response amplitudes relative to baseline are similar between dopamine and FAUC-73 at the moment of antagonist application, despite FAUC-73 behaving as a partial agonist in the concentration-response experiments, as noted above. This is likely due to the faster response kinetics of FAUC-73: The mean FAUC-73-induced response tends rise to faster compared to the dopamine-induced response, which is farther from having reached a steady-state level at the moment of antagonist application (147 s after agonist addition). In contrast, the 15 min incubation used in the concentration-response experiments gives more time for dopamine-induced responses to reach steady-state.

**Fig. 3:**
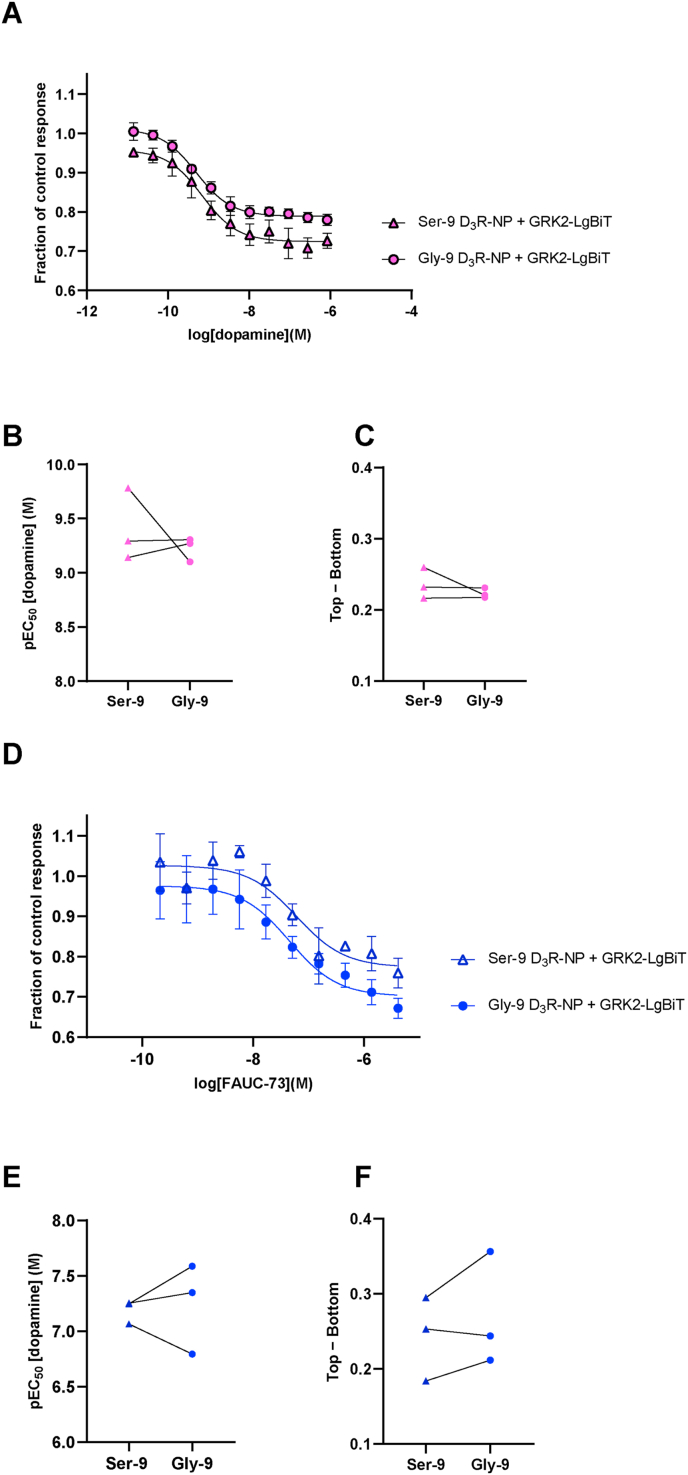
A nanoluciferase complementation assay to monitor GRK2 interactions with the D_3_R in live cells. A) Concentration-response curves for dopamine-induced luminescence increase obtained in cells transfected with Ser-9 or Gly-9 (as indicated) D_3_R–NP constructs and GRK2-LgBiT. Data points represent means ± s.e.m. from three individual experiments performed in octuplicate wells. B) pEC_50_ and C) efficacy expressed as the Top − Bottom parameters fitted to data from each of the three individual experiments, the means of which are shown in A. No significant differences are observed between the Ser-9 and Gly-9 variants (Student's paired *t*-test). D) Concentration-response curves for FAUC-73-induced luminescence increase obtained in cells transfected with Ser-9 or Gly-9 (as indicated) D_3_R-NP constructs and GRK2-LgBiT. Data points represent means ± s.e.m. from three individual experiments performed in quadruplicate wells. E) pEC_50_ and F) efficacy expressed as the Top − Bottom parameters fitted to data from each of the three individual experiments, the means of which are shown in D. No significant differences in pEC_50_ nor Top − Bottom are observed between the Ser-9 and Gly-9 variants (Student's paired *t*-test). See also Supplementary Table S3 for a tabular representation of the data, including s.e.m.

Finally, we directly investigated the interaction between GRK2 and the D_3_R, coexpressing a LgBiT-tagged GRK2 construct along with D_3_R-NP. We have previously verified that the luminescence output from cells expressing this construct alone does not respond to dopamine application [6]. Contrary to correspondingly tagged D_2_R and D_4_R, where dopamine application evoked a signal increase in cells coexpressing GRK2-LgBiT [6], a concentration-dependent signal decrease was observed in these D_3_R-NP-coexpressing cells (Fig. 3A). Apparent competition between GRK2 and β-arrestin2 for binding to the D_3_R has been described [8], and the present results are compatible with such an interpretation. Alternatively, the conformational change associated with D_3_R activation might reduce its affinity for GRK2. Again, there were no significant differences in the concentration-dependence of the dopamine-induced responses in luminescence output between the Ser-9 and Gly-9 variants (Fig. 3B, C and Supplementary Table S3). The effect of FAUC-73 in this assay was similar to that of dopamine, resulting in a concentration-dependent signal decrease that did not differ significantly between the Ser-9 and Gly-9 variants (Fig. 3D–F and Supplementary Table S3).

In agreement with our present findings regarding the effect of GRK2 cotransfection on arrestin recruitment to the D_3_R, Forster et al. [9] also did not find any effect of GRK2 overexpression, nor of the GRK2/3 inhibitor Cmpd101, on the potency of the D_2/3_R agonist quinpirole in promoting arrestin-D_3_R interactions. In addition, we did not observe any differences in arrestin recruitment between the Ser-9 and Gly-9 D_3_R variants. While various reports have described differences in dopamine potency or affinity between these two common variants [16,21], there are also other studies that did not replicate these findings [22]. Our findings do not support any differences in agonist potency nor receptor expression levels between these variants, although the possibility remains that there are signalling or trafficking differences in the native, neuronal environment which are not recapitulated in our artificial expression system. It should also be emphasized that our ability to detect any effects of GRK2 coexpression or of the Ser-9/Gly-9 variant is limited by the variability inherent to our measurements. There is some inter-experimental variation in the pEC_50_ and Top − Bottom parameters (Fig. 1B and C) that may, e.g., be related to our use of transiently transfected cells.

Overall, the agonist-induced luminescence increases observed here are smaller than in our previous experiments with the D_2_R and D_4_R [6]. This may reflect a lower ability of the D_3_R, compared to the D_2_R, to recruit arrestins, as has been previously described [40]. In addition, HEK293 cells are known to prominently express both β-arrestin1 and -2 [41], and it is thus likely that endogenous arrestins compete for binding to the activated receptor, thus reducing the maximum response that can be obtained in our assay. The aforementioned study by Forster et al. [9] used a similar luciferase complementation strategy, but based on emerald luciferase instead of the smaller and brighter nanoluciferase [42]. While they were able to detect arrestin recruitment to the D_2_R in both lysed and intact cells, interaction between the D_3_R and arrestin was observable only following cell lysis. This difference between their findings and the present ones may be due to the difference in brightness of the two luciferases, and/or due to a reduced affinity of arrestin for the activated D_3_R resulting from steric hindrance from the bulkier emerald luciferase. Disruption of the cell membrane presumably allows greater access of the luciferase to its substrate, thus compensating for its relatively lower brightness. Here, we were able to harness the sensitivity of a nanoluciferase complementation assay to obtain time-resolved measurements of arrestin recruitment to the D_3_R in live cells.

## CRediT authorship contribution statement

**Viktor Burström:** Writing – review & editing, Visualization, Methodology, Investigation, Formal analysis, Data curation. **Kuiying Xu:** Writing – review & editing, Resources, Methodology, Investigation. **Emilio Garro-Martínez:** Writing – review & editing, Supervision, Methodology, Investigation. **Robert H. Mach:** Writing – review & editing, Supervision, Resources, Project administration, Methodology, Investigation. **Kristoffer Sahlholm:** Writing – original draft, Visualization, Supervision, Resources, Project administration, Methodology, Investigation, Funding acquisition, Formal analysis, Data curation, Conceptualization. **Nibal Betari:** Writing – review & editing, Writing – original draft, Supervision, Resources, Project administration, Methodology, Investigation, Funding acquisition, Formal analysis, Data curation, Conceptualization.

## Declaration of competing interest

The authors declare that they have no known competing financial interests or personal relationships that could have appeared to influence the work reported in this paper.
