# Supplementary material for: A nanoluciferase complementation-based assay for monitoring β-arrestin2 recruitment to the dopamine D3 receptor
Source: Biochem Biophys Rep. 2025 Apr 18;42:102019. doi: 10.1016/j.bbrep.2025.102019 (PMC12032866; doi:10.1016/j.bbrep.2025.102019)
Supplement: Multimedia component 1 [file mmc1.docx]

| Individual experiments: D_3_R-NP + LgBiT-β-arrestin2 | | | | | |
| --- | --- | --- | --- | --- | --- |
| Experiment number | Variant | pEC_50_ ± s.e.m. (– GRK2) | pEC_50_ ± s.e.m. (+ GRK2) | Top − Bottom ± s.e.m. (– GRK2) | Top − Bottom ± s.e.m. (+ GRK2) |
| 1 | Ser-9 | 9.988 ± 0.111 | 10.215 ± 0.099 | 0.313 ± 0.017 | 0.285 ± 0.014 |
|  | Gly-9 | 10.160 ± 0.086 | 10.029 ± 0.077 | 0.275 ± 0.012 | 0.312 ± 0.012 |
| 2 | Ser-9 | 10.263 ± 0.109 | 10.392 ± 0.113 | 0.227 ± 0.012 | 0.213 ± 0.012 |
|  | Gly-9 | 10.607 ± 0.095 | 10.124 ± 0.096 | 0.171 ± 0.008 | 0.225 ± 0.010 |
| 3 | Ser-9 | 10.087 ± 0.269 | 9.951 ± 0.060 | 0.063 ± 0.008 | 0.314 ± 0.009 |
|  | Gly-9 | 10.201 ± 0.078 | 10.059 ± 0.063 | 0.277 ± 0.010 | 0.386 ± 0.011 |
| 4 | Ser-9 | 10.064 ± 0.092 | 10.005 ± 0.100 | 0.186 ± 0.008 | 0.248 ± 0.012 |
|  | Gly-9 | 10.206 ± 0.099 | 10.050 ± 0.072 | 0.197  ± 0.009 | 0.313 ± 0.011 |

**Supplementary Table S1.** Potencies and efficacies of dopamine in individual nanoluciferase complementation experiments using Ser-9/Gly-9 D_3_R-NP and LgBiT- β-arrestin2 in the absence or presence of exogenous GRK2.
