# Supplementary material for: A nanoluciferase complementation-based assay for monitoring β-arrestin2 recruitment to the dopamine D3 receptor
Source: Biochem Biophys Rep. 2025 Apr 18;42:102019. doi: 10.1016/j.bbrep.2025.102019 (PMC12032866; doi:10.1016/j.bbrep.2025.102019)
Supplement: Multimedia component 2 [file mmc2.docx]

| Experiment number | Variant | Absorbance at 450 nm |
| --- | --- | --- |
| 1 | Ser-9 | 1.612 ± 0.032 |
|  | Gly-9 | 1.494 ± 0.049 |
| 2 | Ser-9 | 0.838 ± 0.021 |
|  | Gly-9 | 0.907 ± 0.040 |
| 3 | Ser-9 | 1.089 ± 0.013 |
|  | Gly-9 | 0.926 ± 0.020 |

**Supplementary Table S2.** Surface expression measured as absorbance at 450 nm in a whole-cell ELISA assay for Ser-9/Gly-9 D_3_R-NP.
