# Supplementary material for: A nanoluciferase complementation-based assay for monitoring β-arrestin2 recruitment to the dopamine D3 receptor
Source: Biochem Biophys Rep. 2025 Apr 18;42:102019. doi: 10.1016/j.bbrep.2025.102019 (PMC12032866; doi:10.1016/j.bbrep.2025.102019)
Supplement: Multimedia component 3 [file mmc3.docx]

| Individual experiments: D_3_R-NP + GRK2-LgBiT | | | |
| --- | --- | --- | --- |
| Experiment number | Variant | pEC_50_ ± s.e.m. | Top − Bottom ± s.e.m. |
| Agonist: Dopamine | | | |
| 1 | Ser-9 | 9.405 ± 0.099 | 0.260 ± 0.014 |
|  | Gly-9 | 9.328 ± 0.117 | 0.221 ± 0.013 |
| 2 | Ser-9 | 9.147 ± 0.138 | 0.232 ± 0.016 |
|  | Gly-9 | 9.277 ± 0.163 | 0.231 ± 0.019 |
| 3 | Ser-9 | 8.983 ± 0.125 | 0.216 ± 0.013 |
|  | Gly-9 | 9.332 ± 0.161 | 0.217 ± 0.018 |
| Agonist: FAUC-73 | | | |
| 1 | Ser-9 | 7.248 ± 0.371 | 0.184 ± 0.033 |
|  | Gly-9 | 7.588 ± 0.217 | 0.212 ± 0.021 |
| 2 | Ser-9 | 7.066 ± 0.397 | 0.253 ± 0.050 |
|  | Gly-9 | 6.794 ± 0.375 | 0.244 ± 0.049 |
| 3 | Ser-9 | 7.254 ± 0.219 | 0.295 ± 0.031 |
|  | Gly-9 | 7.350 ± 0.161 | 0.356 ± 0.027 |

**Supplementary Table S3.** Potencies and efficacies of dopamine and FAUC-73 in individual nanoluciferase complementation experiments using Ser-9/Gly-9 D_3_R-NP and GRK2-LgBiT.
