# Supplementary material for: A nanoluciferase complementation-based assay for monitoring β-arrestin2 recruitment to the dopamine D3 receptor
Source: Biochem Biophys Rep. 2025 Apr 18;42:102019. doi: 10.1016/j.bbrep.2025.102019 (PMC12032866; doi:10.1016/j.bbrep.2025.102019)
Supplement: Multimedia component 4 [file mmc4.docx]

**Supplementary Figure S1.** Effect of agonists on luminescence in cells expressing D_3_R-NP or LgBiT-β-arrestin2 separately. A) Luminescence intensities (as recorded by the plate reader, expressed as arbitrary luminescence units; ALU) from cells transfected separately with either Ser-9 D_3_R-NP, Gly-9 D_3_R-NP, or LgBiT-β-arrestin2 and treated with vehicle (HBSS), 1 µM dopamine, or 4.2 µM FAUC-73, as indicated. B) Luminescence in agonist-treated cells normalized to vehicle control (same data as in A). Data points represent means ± s.e.m. from three individual experiments performed in quadruplicate wells.
