# Supplementary figures and images for: A nanoluciferase complementation-based assay for monitoring β-arrestin2 recruitment to the dopamine D3 receptor
Source: Biochem Biophys Rep. 2025 Apr 18;42:102019. doi: 10.1016/j.bbrep.2025.102019 (PMC12032866; doi:10.1016/j.bbrep.2025.102019)

**A**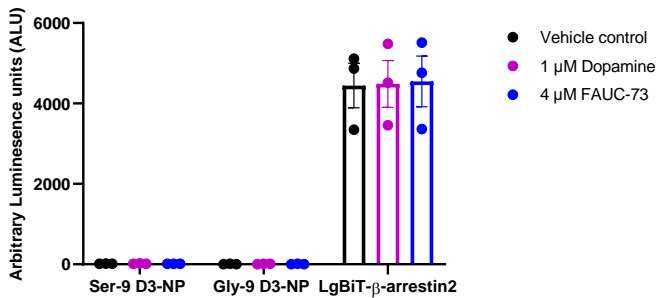**B**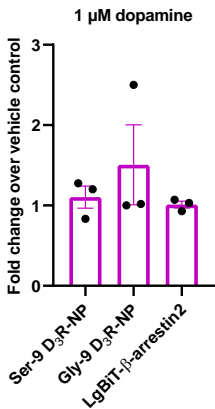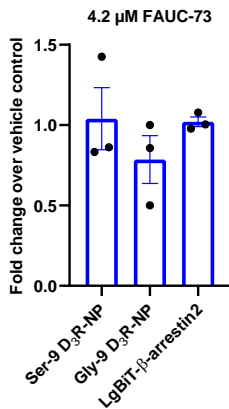

Supplement: Multimedia component 5 [file mmc5.pdf]

### Ser-9

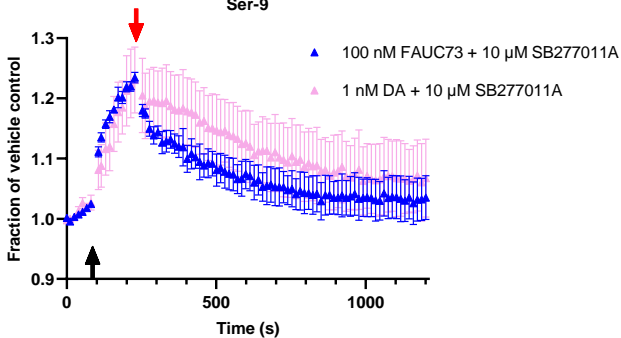

### Gly-9

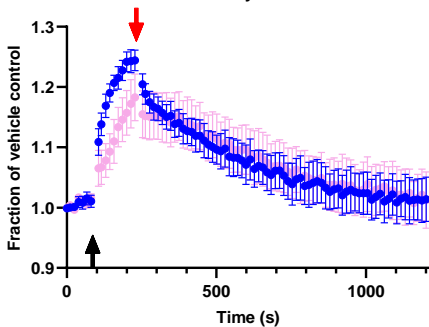

Supplement: Multimedia component 7 [file mmc7.pdf]
