# Supplementary material for: A nanoluciferase complementation-based assay for monitoring β-arrestin2 recruitment to the dopamine D3 receptor
Source: Biochem Biophys Rep. 2025 Apr 18;42:102019. doi: 10.1016/j.bbrep.2025.102019 (PMC12032866; doi:10.1016/j.bbrep.2025.102019)
Supplement: Multimedia component 6 [file mmc6.docx]

**Supplementary Figure S2.** Time-resolved responses to 100 nM FAUC-73 or 1 nM dopamine (black arrow), followed by the application of 10 µM SB277011A (red arrow), in cells coexpressing Ser-9 (above) or Gly-9 (below) D_3_R-NP with LgBiT-βarrestin2. Data represent means ± s.e.m. from four individual experiments performed in octuplicate wells. Same data sets as in Figure 2B, normalized only to baseline (first data point in the time series).
